# Mutation in the *PTEN/MMAC1* gene in archival low grade and high grade gliomas

**DOI:** 10.1038/sj.bjc.6690246

**Published:** 1999-03

**Authors:** M P A Davies, F E M Gibbs, N Halliwell, K A Joyce, M M Roebuck, M L Rossi, J Salisbury, D R Sibson, L Tacconi, C Walker

**Affiliations:** JK Douglas Cancer Research Laboratory, Clatterbridge Hospital, Bebington, Merseyside, L63 4JY, UK; The Walton Centre for Neurology and Neurosurgery, Walton Hospital, Liverpool, L9 1AE, UK

**Keywords:** *PTEN*, *MMAC1*, glioma, mutation, paraffin-embedded tissue

## Abstract

The *PTEN* gene, located on 10q23.3, has recently been described as a candidate tumour suppressor gene that may be important in the development of advanced cancers, including gliomas. We have investigated mutation in the *PTEN* gene by direct sequence analysis of PCR products amplified from samples microdissected from 19 low grade (WHO Grade I and II) and 27 high grade (WHO grade III and IV) archival, formalin-fixed, paraffin-embedded gliomas. Eleven genetic variants in ten tumours have been identified. Eight of these are DNA sequence changes that could affect the encoded protein and were present in 0/2 pilocytic astrocytomas, 0/2 oligoastrocytomas, 0/1 oligodendroglioma, 0/14 astrocytomas, 3/13 (23%) anaplastic astrocytomas and 5/14 (36%) glioblastomas. *PTEN* mutations were found exclusively in high grade gliomas; this finding was statistically significant. Only two of the *PTEN* genetic variants have been reported in other studies; two of the genetic changes are in codons in which mutations have not been found previously. The results of this study indicate that mutation in the *PTEN* gene is present only in histologically more aggressive gliomas, may be associated with the transition from low histological grade to anaplasia, but is absent from the majority of high grade gliomas. © 1999 Cancer Research Campaign


British Journal of Cancer (1999) **79**(9/10), 1542–1548
© 1999 Cancer Research Campaign
Article no. bjoc.1998.0246

### Mutation in the *PTEN/MMAC1* gene in archival low grade and high grade gliomas

**MPA Davies[1], FEM Gibbs[1], N Halliwell[1], KA Joyce[1], MM Roebuck[1], ML Rossi[2]*, J Salisbury[1], DR Sibson[1], L Tacconi[2] and C Walker[1]**

[1]JK Douglas Cancer Research Laboratory, Clatterbridge Hospital, Bebington, Merseyside L63 4JY, UK; [2]The Walton Centre for Neurology and Neurosurgery, Walton Hospital, Liverpool L9 1AE, UK

**Summary** The *PTEN* gene, located on 10q23.3, has recently been described as a candidate tumour suppressor gene that may be important in the development of advanced cancers, including gliomas. We have investigated mutation in the *PTEN* gene by direct sequence analysis of PCR products amplified from samples microdissected from 19 low grade (WHO Grade I and II) and 27 high grade (WHO grade III and IV) archival, formalin-fixed, paraffin-embedded gliomas. Eleven genetic variants in ten tumours have been identified. Eight of these are DNA sequence changes that could affect the encoded protein and were present in 0/2 pilocytic astrocytomas, 0/2 oligoastrocytomas, 0/1 oligodendroglioma, 0/14 astrocytomas, 3/13 (23%) anaplastic astrocytomas and 5/14 (36%) glioblastomas. *PTEN* mutations were found exclusively in high grade gliomas; this finding was statistically significant. Only two of the *PTEN* genetic variants have been reported in other studies; two of the genetic changes are in codons in which mutations have not been found previously. The results of this study indicate that mutation in the *PTEN* gene is present only in histologically more aggressive gliomas, may be associated with the transition from low histological grade to anaplasia, but is absent from the majority of high grade gliomas.

**Keywords:** *PTEN*; *MMAC1*; glioma; mutation; paraffin-embedded tissue

Gliomas constitute 40–67% of primary neoplasms of the central nervous system. Despite advances in therapy, the prognosis for the most malignant gliomas remains poor with post-treatment survival times of often less than a year (Black, 1991; Hildebrand et al, 1997). Low grade gliomas are associated with longer median survival, but a proportion progress to anaplastic tumours or glioblastomas accompanied by sequential acquisition of genetic alterations (Black, 1991; Ohgaki et al, 1995; Louis, 1997). While many of the molecular genetic events involved in the histogenesis of gliomas have already been characterized (reviewed in Louis, 1997), identification of those events associated with the transition of astrocytic tumours from low grade to higher grade is important in the quest for improved diagnosis and future therapeutic advances.

Loss of all or part of chromosome 10q occurs in approximately 70% of glioblastomas but only rarely in low grade gliomas (Bigner et al, 1988; Steck et al, 1995; Rasheed et al, 1995). On the basis of cytogenetic and loss of heterozygosity (LOH) studies, one or more tumour suppressor genes involved in the development of advanced cancers has been postulated within chromosome 10q (Bigner et al, 1988; Karlbom et al, 1993; Steck et al, 1995; Rasheed et al, 1995; Sonoda et al, 1996; Böstrom et al, 1998). Recent investigations have led to the isolation of a candidate tumour suppressor gene, *PTEN* (*MMAC1*), located at chromosome 10q23.3 (Li et al, 1997; Steck et al, 1997), which encodes a protein tyrosine phosphatase (Li and Sun, 1997) and has homology to tensin and auxilin (Li et al, 1997; Myers and Tonks, 1997; Steck et al, 1997). Germ-line mutations in the *PTEN* gene have been found in a variety of inheritable disorders associated with formation of multiple benign tumours and an increased incidence of malignant disease (Liaw et al, 1997; Lynch et al, 1997; Marsh et al, 1997; Nelen et al, 1997). LOH at the *PTEN* locus and mutations in the *PTEN* gene have been reported in a wide variety of sporadic cancers, including prostate (Cairns et al, 1997; Suzuki et al, 1998), primary breast tumours (Rhei et al, 1997), malignant melanomas (Guldberg et al, 1997) and endometrial carcinomas (Tashiro et al, 1997; Risinger et al, 1997). In the gliomas investigated so far, 16% of anaplastic astrocytomas and 26% of glioblastomas had *PTEN* mutations, but none have been found in low grade gliomas (Li et al, 1997; Liu et al, 1997; Rasheed et al, 1997; Sakurada et al, 1997; Steck et al, 1997; Wang et al, 1997; Böstrom et al, 1998; Chiariello et al, 1998). Thus, genetic alterations in the *PTEN* gene may be involved in the progression of gliomas to advanced disease.

Investigations of mutation in the *PTEN* gene in gliomas have so far been restricted to DNA or RNA extracted from snap frozen tumour. The results obtained from such studies may not correlate precisely with histology and are inappropriate for the tiny amounts of processed biopsy material on which diagnosis is often based. Microdissection of regions of known histology increases the neoplastic cell population in the samples for analysis and allows mutation to be related accurately to histological grade. This technique can be applied to diagnostic biopsy material.

In order to investigate further the relationship of mutation in the *PTEN* gene to the histological grade of gliomas, we have developed methods for *PTEN* mutation detection by direct sequence analysis in samples microdissected from archival, formalin-fixed, paraffin-embedded tissues. We have investigated the incidence of mutation in the *PTEN* gene in a series of 19 low grade [World

*Received 27 March 1998*
*Revised 27 May 1998*
*Accepted 3 June 1998*

*Correspondence to:* C Walker

*Affiliation: International University of Catalonia (Barcelona), Spain.

**Table 1** Clinical information

| Case number | Gender | Age at diagnosis | Location | Prior therapy | Survival (months) | WHO classification | WHO grade | Sequence variant |
|---|---|---|---|---|---|---|---|---|
| 1 | Male | 16 | Supratentorial | No | 144+ | Astrocytoma | II | No |
| 2 | Male | 65 | Supratentorial | No | # | Astrocytoma | II | No |
| 3 | Male | 68 | Supratentorial | No | 39 | Astrocytoma | II | No |
| 4 | Female | 61 | Supratentorial | No | 25 | Astrocytoma | II | No |
| 5 | Female | 53 | Supratentorial | No | 56+ | Astrocytoma | II | No |
| 6 | Female | 40 | Supratentorial | No | 34+ | Astrocytoma | II | No |
| 7 | Female | 48 | Supratentorial | No | 29+ | Astrocytoma | II | No |
| 8 | Female | 45 | Supratentorial | No | 30+ | Astrocytoma | II | No |
| 9 | Male | 30 | Supratentorial | No | 20 | Astrocytoma | II | No |
| 10 | Male | 37 | Supratentorial | No | 25+ | Astrocytoma | II | No |
| 11 | Male | 39 | Supratentorial | No | 17+ | Astrocytoma | II | No |
| 12 | Male | 68 | Supratentorial | No | 14 | Astrocytoma | II | No |
| 13 | Male | 55 | Supratentorial | No | 20+ | Astrocytoma | II | No |
| 14 | Female | 13 | Infratentorial | No | 38+ | Astrocytoma (pilocytic) | I | No |
| 15 | Female | 57 | Infratentorial | No | 33+ | Astrocytoma (pilocytic) | I | No |
| 16 | Female | 7 | Supratentorial | Yes | 42+ | Astrocytoma | II | No |
| 17 | Female | 33 | Supratentorial | No | 58+ | Oligoastrocytoma | II | No |
| 18 | Female | 26 | Supratentorial | No | 19+ | Oligoastrocytoma | II | No |
| 19 | Male | 64 | Supratentorial | No | 37+ | Oligodendroglioma | II | No |
| 20 | Male | 23 | Supratentorial | No | 15 | Anaplastic astrocytoma | III | No |
| 21 | Female | 41 | Supratentorial | No | 13 | Anaplastic astrocytoma | III | Yes |
| 22 | Female | 73 | Supratentorial | No | 3 | Anaplastic astrocytoma | III | No |
| 23 | Male | 62 | Supratentorial | No | 2 | Anaplastic astrocytoma | III | Yes |
| 24 | Male | 66 | Supratentorial | No | 1 | Anaplastic astrocytoma | III | No |
| 25 | Male | 71 | Supratentorial | No | 12 | Anaplastic astrocytoma | III | No |
| 26 | Male | 44 | Supratentorial | No | 33+ | Anaplastic astrocytoma | III | No |
| 27 | Female | 48 | Supratentorial | No | 27+ | Anaplastic astrocytoma | III | No |
| 28 | Male | 60 | Supratentorial | No | 12+ | Anaplastic astrocytoma | III | No |
| 29 | Male | 50 | Supratentorial | No | 12 | Anaplastic astrocytoma | III | Yes |
| 30 | Male | 51 | Supratentorial | No | 1 | Anaplastic astrocytoma | III | Yes |
| 31 | Female | 32 | Supratentorial | No | 46+ | Anaplastic astrocytoma* | III | No |
| 32 | Male | 45 | Supratentorial | No | 0.2 | Anaplastic astrocytoma* | III | No |
| 33 | Female | 62 | Supratentorial | No | 14 | Glioblastoma | IV | No |
| 34 | Female | 76 | Supratentorial | No | # | Glioblastoma | IV | No |
| 35 | Female | 61 | Supratentorial | No | 11 | Glioblastoma | IV | Yes |
| 36 | Female | 79 | Supratentorial | No | 5 | Glioblastoma | IV | No |
| 37 | Female | 59 | Supratentorial | No | 10 | Glioblastoma | IV | No |
| 38 | Female | 36 | Supratentorial | No | 12 | Glioblastoma | IV | Yes |
| 39 | Male | 62 | Supratentorial | No | 2 | Glioblastoma | IV | No |
| 40 | Female | 65 | Supratentorial | No | 1 | Glioblastoma | IV | Yes |
| 41 | Female | 66 | Supratentorial | No | 13 | Glioblastoma | IV | Yes |
| 42 | Female | 43 | Supratentorial | No | 9 | Glioblastoma | IV | Yes |
| 43 | Male | 49 | Supratentorial | No | 19 | Glioblastoma | IV | Yes |
| 44 | Male | 27 | Supratentorial | No | 7 | Glioblastoma** | IV | No |
| 45 | Male | 57 | Supratentorial | Yes | 22 | Glioblastoma | IV | No |
| 46 | Male | 35 | Supratentorial | Yes | 35 | Glioblastoma | IV | No |

*Gemistocytic; **giant cell. Survival details: + = patient alive, # = patient lost to follow-up.

Health Organization (WHO) grades I and II] and 27 high grade gliomas (13 anaplastic astrocytomas WHO grade III, 14 glioblastomas WHO grade IV) and relate our findings to the histology of the samples analysed.

### MATERIALS AND METHODS

### Tumour samples

Formalin-fixed paraffin-embedded blocks of gliomas, diagnosed from 1986 to 1997, were obtained from the archives at the Neurosurgery Centre, Walton Hospital, Liverpool, UK. Cases selected for study were either biopsies or surgical resections, had clinical information available, and had both tumour and normal brain tissue present for analysis. None of the patients had chemotherapy prior to surgery or biopsy. The histological diagnosis of all cases was re-reviewed (MLR) and, where necessary, immunocytochemistry using antibodies to GFAP, α actin, Factor VIII and CD 34 was carried out to assist accurate histopathological diagnosis. Tumours were classified according to WHO criteria (Kleihues et al, 1993).

### Microdissection

Microdissection was used to enrich the population of either normal or tumour cells for analysis. Prior to microdissection, a 5 μm serial section stained with haematoxylin and eosin was examined by a pathologist (MLR) and regions of tissue selected for analysis. The

© Cancer Research Campaign 1999

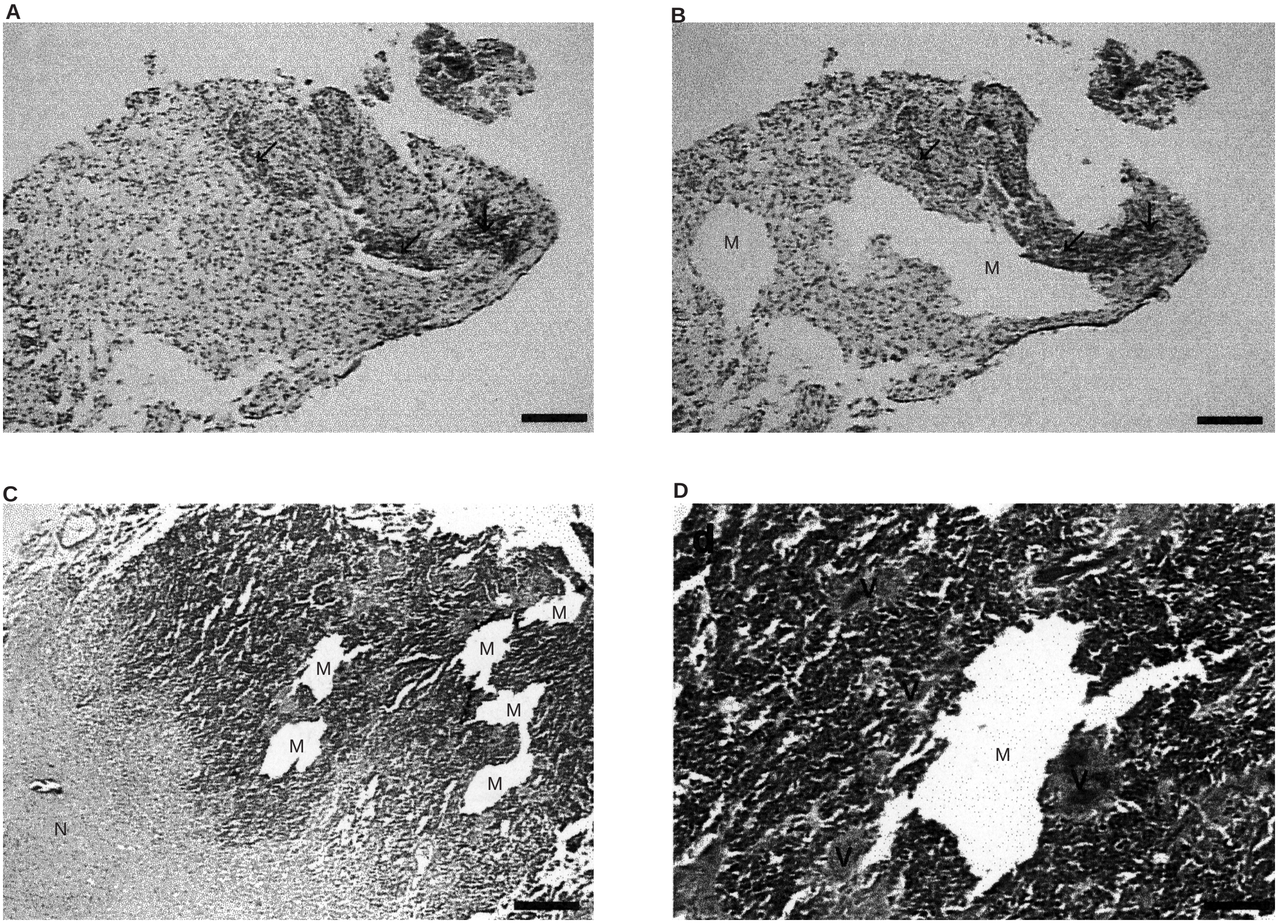

**Figure 1** Microdissection of samples for mutation analysis. (**A, B**) Case 1 – an astrocytoma WHO grade II; (**A**) haematoxylin and eosin stain of 5 μM serial section not microdissected (bar = 500 μM); (**B**) haematoxylin and eosin stain of 10 μM section after microdissection (bar = 500 μM). (**C, D**) Case 20 – an anaplastic astrocytoma WHO grade III; (**C**) hematoxylin and eosin stain of 10 μm section after microdissection (bar = 1 mm); (**D**) same section at higher magnification illustrating microdissection to avoid vessels (bar = 250 μm). Illustrations are video captured computer generated images. M – tissue area microdissected; V – vascular proliferation N – normal tissue with infiltrating tumour; arrow – region of tissue containing blood vessels marked slide was used to guide microdissection of the chosen histological regions from three serial 10 μM sections, dewaxed through xylene and graded alcohols to water and stained with 0.1% toluidine blue. Microdissection was carried out using a stereo-dissecting microscope and an electrolytically polished tungsten needle controlled by a micromanipulator (Going et al, 1996). Tissue fragments of 0.01–0.15 $mm^2$ were microdissected avoiding, where possible, blood vessels and regions of necrosis. These fragments were collected in 12.5-μl buffer (10 mM Tris-HCl pH 8.0, 1 mM EDTA). Multiple fragments (up to 15 samples) within the same low power field (× 50) were pooled and digested at 37°C for 72 h following the addition of an equal volume of buffer containing 2 mg $ml^{-1}$ proteinase K and 2.0% Tween 20. This procedure created a tissue extract of up to 300 μl representing 0.002–0.02 $mm^2$ of tissue $\mu l^{-1}$. Proteinase K was inactivated by boiling for 15 min. Extracts were stored at –20°C. Digitized computer images were archived before and after microdissection. These images and haematoxylin and eosin stains of the microdissected sections and serial sections were used for pathological review.

### PCR amplification

Polymerase chain reaction (PCR) amplification was performed in 20-μl or 40-μl reaction volumes for first round or second round reactions, respectively, containing 1 × amplitaq PCR buffer, 200 μM each dNTP, 1 μM each primer (designed for use with paraffin-embedded tissues using Oligo software) and 0.1 units Amplitaq Gold (Perkin Elmer). Two-microlitre aliquots of tissue extract were used as target in first round reactions and 2 μl of the first round reaction were taken into second round reactions. 1.5 mM magnesium chloride ($MgCl_2$) was used for amplification of exons 1, 2 and 8b, while 2.5 mM $MgCl_2$ was used for exon 8a. All other *PTEN* exons required 2 mM $MgCl_2$. For some samples, nested reverse primers for exons 4 and 5a were used in the second round. *PTEN* exons were amplified with the following PCR conditions: –94°C for 10 min followed by 35 cycles (exons 1, 2, 3, 4, 5b, 6, 7, 8b) or 38 cycles (exons 5a, 8a, 9) of 94°C for 30 s, 56°C for 30 s, 72°C for 30 s, then 72°C extension for 10 min. In the second round the same cycling conditions were used but for 35–40 cycles.

 © Cancer Research Campaign 1999

**Table 2** Primers for PCR amplification of the *PTEN* gene from formalin-fixed, paraffin-embedded tissues

| Exon | Forward primer | Reverse primer |
|---|---|---|
| 1 | caagtccagagccatttcc | cccaacgttctaagagagtga |
| 2 | ttcttttagtttgattgctg | gtatctttttctgtggcttag |
| 3 | ctgtcttttggtttttctt | caagcagataactttcactta |
| 4 | tataaagattcaggcaatgtt | ctcgataatctggatgactc |
| | | *cagtctatcgggtttaagtta |
| 5a | ttgttaattaaaaattcaagag | gcacatatcattacaccagt |
| | | *cctttccagctttacagt |
| 5b | tgaccaatggctaagtgaa | aaaagaaacccaaaatctgtt |
| 6 | cccagttaccatagcaat | taagaaaactgttccaataca |
| 7 | ttgacagttaaaggcatttc | cttattttggatatttctccc |
| 8a | ttcatttcttttcttttcttt | ggttggctttgtctttatt |
| 8b | ccaggaccagaggaaac | cacatacatacaagtcaccaa |
| 9 | agtcatatttgtgggtttt | ttattttcatggtgttttatc |

*Nested primers.

### Mutation detection

Sequence analysis of PCR products was carried out blind, without knowledge of the histological grading of the samples being analysed. The nucleotide sequence of each PCR product was determined in the forward and reverse directions by cycle-sequencing using dRhodamine dye-terminator kits (ABI) with the primers used for PCR. Sequencing reactions were purified by ethanol precipitation prior to electrophoresis using an ABI 377. Sequence data analysis used Factura and Sequence Navigator software (ABI). Sequence variations detected in both forward and reverse directions were confirmed by analysis of a second independent PCR product amplified from the same tissue extract. DNA amplified from normal brain tissue was analysed to determine whether the observed sequence variation was acquired.

### Microsatellite analysis

Normal and tumour tissue extracts were analysed for allelic imbalance in chromosome 10q using polymorphic markers D10S1687, D10S583, AFMA086WG9 and D10S2491 (Cairns et al, 1997; Rasheed et al, 1997; Steck et al, 1997). Two microlitres of tissue extract was used in 10-μl PCR reactions containing 1 × amplitaq PCR buffer, 200 μM each dNTP, 1.5 mM $MgCl_2$ or [2 mM $MgCl_2$ for D10S1687], 0.1 μM each primer and 0.05 units Amplitaq Gold (Perkin Elmer). PCR conditions were: –94°C for 10 min followed by 48 cycles of 94°C for 30 s, 56°C [or 60°C for D10S1687] for 30 s, 72°C for 30 s, followed by 72°C extension for 10 min. The 5´ ends of reverse primers were labelled with fluorescent FAM. One microlitre of PCR products was mixed with 0.33 μl ROX-350 size standard (Perkin Elmer) and 2 μl formamide-loading buffer. Samples were denatured immediately prior to gel loading by heating to 96°C for 5 min, followed by snap-chilling in an ice-water bath. Analysis was performed using denaturing (6 M urea) 5.5% acrylamide gels on an ABI-377 Fluorescent DNA Analyser and loading 0.75 μl per lane. Results were analysed using GeneScan software. In informative cases, allelic loss was scored if the area under one allele peak was reduced to less than 30% of its value in the normal tissue extract (relative to the other allele).

### Statistical analysis

Data were analysed using Fisher–Irwin Exact test.

### RESULTS

Clinical information for the cases analysed in this study is given in Table 1. The cases studied were predominantly adult, astrocytic, supratentorial tumours obtained prior to therapy (13 low grade and 25 high grade). For tumour samples, the areas chosen for microdissection were most representative of the histological grading of the tumour. Microdissected samples from anaplastic astrocytomas and glioblastomas were judged, by a pathologist, to be > 80% and > 90% tumour cells, respectively. Tumour samples from astrocytomas and other low grade tumours were as pure as could be achieved from the tissues available. Samples analysed from all low grade cases were considered to contain approximately 80% tumour cells, except those from cases 2 and 3 which contained approximately 20–30% and 50% neoplastic cells, respectively. For normal tissue, normal brain most free of infiltrating tumour cells was selected. Figure 1 shows examples of microdissection.

Previous studies, involving amplification of the β-globin gene from microdissected samples (< 1 $mm^2$) isolated from a series of archival brain tumours, from the Neurosurgery Centre, Walton Hospital, Liverpool, UK, have shown that PCR amplification was more efficient with amplicons less than 275 bp. *PTEN* primers for amplicons < 275 bp were designed (Table 2) from the sequence obtained by amplifying human placental genomic DNA using the

**Table 3** PTEN sequence variants

| Case | Codon | Exon | Base* | Change | Effect | Acquired |
|---|---|---|---|---|---|---|
| 21 | 76 | 4 | 226–228 | Del TAT | Delete Tyr | YES |
| 23 | 173 | 6 | 519 | G to A | Arg to His | YES |
| 29 | Non-coding | Intron 2 | Exon 3–34 | A to G | None? | ND |
| 30 | 131 | 5 | 392 | C to A | Thr to Asn | YES |
| 35 | Non-coding | Intron 3 | Exon 3 +5 | G to A | Splice mutation | YES |
| 38 | Non-coding | Intron 3 | Exon 4–39 | A to G | None? | NO |
| 38 | 98 | 5 | 294 | A to G | Silent | ND |
| 40 | ? | 5 | ? | Deletion/insertion | Deletion/insertion | YES |
| 41 | 27 | 2 | 80 | A to C | Tyr to Ser | YES |
| 42 | 171 | 6 | 511 | C to T | Gln to stop | YES |
| 43 | 42 | 2 | 125 | T to C | Leu to Pro | YES |

*Numbering of nucleotides according to Li et al 1997 (GenBank accession no. U93051). ND = not determined.

© Cancer Research Campaign 1999

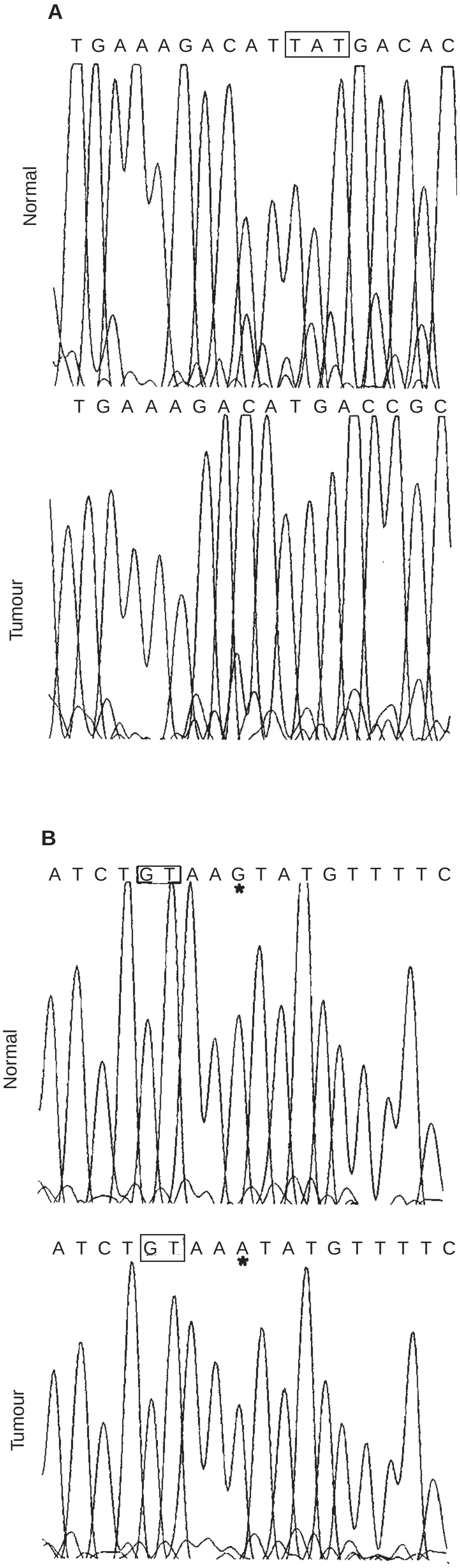

**Figure 2** Sequence data for case 21 deletion (**A**) and case 35 substitution (**B**). In (**A**) the boxed bases are those deleted. In (**B**) the boxed bases are the donor splice site of exon 3; *marks the substituted base

**Table 4** Mutation in the tumours in this series ($n$ = 46) grouped according to histopathological grade

| | Mutation | No mutation | |
|---|---|---|---|
| Gliomas | | | |
| WHO grades I and II (low grade) | 0 | 19 | |
| | | | $P^{\#}$ = 0.014 [0.015*] |
| WHO grades III and IV (high grade) | 8 | 19 | |
| Astrocytic gliomas | | | |
| WHO grades I and II (low grade) | 0 | 16 | |
| | | | $P^{\#}$ = 0.0175 [0.035*] |
| WHO grades III and IV (high grade) | 8 | 19 | |
| Adult astrocytic gliomas | | | |
| WHO grades I and II (low grade) | 0 | 14 | |
| | | | $P^{\#}$ = 0.035 [0.04*] |
| WHO grades III and IV (high grade) | 8 | 19 | |

#*P*-values by Fisher–Irwin exact test (two-tailed probabilities). Because of the limited sensitivity associated with direct sequence analysis, mutation, if present, in samples with < 50% tumour cells may not be detected. Probabilities marked* have been calculated omitting the two low grade tumours with a neoplastic cell population of 50% or less.

*PTEN* primer set published by Steck et al (1997). For analysis of the *PTEN* gene, 11 PCR reactions were required to cover the coding sequence of the nine exons.

Direct sequence analysis of *PTEN* PCR products amplified from microdissected tissue samples was successful for all cases investigated. Sequence data were obtained for 100% of exon sequence and flanking splice sites in all 46 cases. Results obtained are shown in Tables 1 and 3, and in Figure 2.

Of the 11 confirmed genetic variants described in ten tumours, eight were acquired by the tumour, being absent from the normal tissue. Additionally, these mutations are likely to affect the encoded protein (Table 3); four are missense mutations, one is an in-frame single codon deletion, one is a nonsense mutation, one is a non-coding, intronic mutation proximal to the splice site, and one (in case 40) is a putative deletion involving part of exon 5/intron 5. In this case, we were unable to amplify the 3′ part of exon 5 using a primer within exon 5 (for which the priming site has been shown to be present) and all combinations of exon 5 primers given in Table 2 or in Steck et al (1997). All other *PTEN* PCR products amplified successfully from the same DNA preparation, and the missing PCR product, were amplified from normal tissue taken from the same section. Two tumours showed non-coding intronic sequence variations, one of which occurred in both tumour and normal tissue. This tumour also showed a sequence variation resulting in a silent codon change (Table 3).

To determine whether the *PTEN* sequence variations detected in these gliomas were associated with loss of heterozygosity in chromosome 10q, microsatellite marker analysis using markers D10S1687, D10S583, AFMA086WG9 and D10S2491 was carried out. All of the cases with genetic variants of *PTEN* showed allelic loss at all informative loci tested.

In this glioma series, 11 genetic variants have been found; four in the 13 anaplastic astrocytomas, of which three were acquired

 *© Cancer Research Campaign 1999*

and could affect the encoded protein, and seven in the 14 glioblastomas, of which five were acquired and could affect the encoded protein. No mutations were detected in low grade gliomas. *PTEN* mutations were found significantly more often in high grade gliomas compared with low grade gliomas, or low grade adult astrocytic tumours (Table 4).

### DISCUSSION

This study is the first report of analysis of mutation in the recently discovered *PTEN* tumour suppressor gene in microdissected, archival tissues. We have identified eight acquired mutations that could affect the encoded protein in 0/2 pilocytic astrocytomas, 0/2 oligoastrocytomas, 0/1 oligodendroglioma, 0/14 astrocytomas, 3/13 (23%) anaplastic astrocytomas and 5/14 (36%) glioblastomas. Six of these have not been reported previously.

All eight mutations were within the region of highest homology between the *PTEN* gene and the genes for tensin and auxilin. In contrast to previous reports of mainly nonsense or frameshift mutations in this region, resulting in truncation of PTEN protein (Parsons, 1998), we have found predominantly missense substitutions. Only one of the mutations, a missense substitution, was found in the putative phosphatase domain of *PTEN*.

As found for the majority of other *PTEN* mutations, DNA sequence changes that could affect the encoded PTEN protein were only found in association with allelic loss of chromosome 10q. Most of the mutations in this study are single base substitutions. Two of these are in codons not previously shown to be mutated and two are in a region spanning codons 170–173. There are already 12 reported mutations in this region, seven of which were seen in glioblastomas, suggesting that this region may be a mutational ‘hot-spot’. Only one mutation reported here, in case 35, is believed to result in altered splicing of the *PTEN* mRNA. This substitution of A for G, five bases downstream of exon 3, has been reported previously in one Cowdens syndrome family (Marsh et al, 1998). Furthermore, a similar splice site mutation has been found in intron 4 of *PTEN* in another glioblastoma (Böstrom et al, 1998). Mutation or deletion at this position resulted in deletion of exon 4, causing a frameshift-induced termination (Böstrom et al, 1998).

In contrast to other studies of the *PTEN* gene in gliomas, direct sequence analysis of PCR products from microdissected tissue is the most precise method for mutation detection, is associated with fewer false negatives than mutation screening methods (Nollau and Wagener, 1997), increases the sensitivity of detection by enrichment of the neoplastic cell population and enables molecular genetic changes to be related precisely to histology. However, this technique does not allow detection of extensive homozygous deletions, which have been reported to be present in 3–12% of high grade gliomas (Wang et al, 1997; Böstrom et al, 1998), or mutation in minor sub-populations of cells. In this study, mutation, if present in the majority of tumour cells, would be detected in all but two of the cases analysed. The two exceptions were from low grade, poorly cellular tumours.

Although mutation in the *PTEN* gene has now been investigated in over 250 glioblastomas, *PTEN* mutations have so far only been reported in three out of 19 anaplastic astrocytomas (Liu et al, 1997; Rasheed et al, 1997; Chiariello et al, 1998). Combining our results with other studies gives an incidence of *PTEN* mutation in anaplastic astrocytomas of 19% (6/32). Other investigations of *PTEN* mutation in gliomas, which used snap-frozen tissue for analysis and preselection by LOH in chromosome 10q and/or mutation screening methods, reported *PTEN* mutation 9–30% of glioblastomas (Liu et al, 1997, Rasheed et al, 1997; Sakurada et al, 1997, 1998; Steck et al, 1997; Böstrom et al, 1998; Chiariello et al, 1998). The incidence of *PTEN* mutation in the glioblastomas analysed in this study was similar to that reported by Wang et al (1997) who also used direct sequence analysis but without microdissection. Thus, for the majority of gliomas, progression to high grade occurs in the absence of mutation in the *PTEN* gene. However, in our study, as in many others, alternative mechanisms of inactivation of the *PTEN* gene, such as homozygous deletion or mutation in promotor sequences or methylation, have still to be addressed.

In this study *PTEN* mutations were not detected in low grade gliomas. In two of the high grade cases with *PTEN* mutation, low grade histological features were present within the same section. In one case, the low grade tissue was well demarcated from the high grade region, in the second, low grade tissue was in close proximity to a transitional region and the tumour was highly infiltrative. In initial experiments, we have been unable to detect mutation in samples microdissected from low grade areas in the former. Mutation was absent or present only to a minor extent in the low grade samples from the latter (data not given). These observations support the hypothesis that mutation in the *PTEN* gene may be related to the transition from low grade to higher grade. We are currently investigating further the association of mutation in the *PTEN* gene with histology in these cases.

Of the cases that had *PTEN* mutations, all but one were initially classified as either anaplastic astrocytomas or glioblastomas. However, one case with *PTEN* mutation, a biopsy with only a small amount of tissue available, showed some mitosis, no necrosis and minimal capillary endothelial hyperplasia. Histopathological re-review placed this tumour as ‘lower end of the anaplastic scale’, indicating that mutation in the *PTEN* gene can be present in tissues featuring subtle anaplastic changes. Histopathological grading of gliomas is well recognized to be difficult for some cases, especially when diagnosis is based on small amounts of biopsy material which may not be representative of the tumour as a whole. Molecular genetic analysis for mutation in genes such as *PTEN* may prove to be diagnostically useful in the future.

This study was designed to investigate the relationship of *PTEN* mutation to histology; survival information is, however, available for the majority of cases investigated. In this very limited series, although the eight patients with high grade tumours with *PTEN* mutations likely to affect the encoded protein had shorter survival than 17 patients with high grade tumours without *PTEN* mutation, log rank analysis of the Kaplan–Meier plot showed no significant difference ($P = 0.139$). Only one other study has reported survival information for gliomas, also with limited conclusions because of low patient numbers. Rasheed et al (1997) reported no obvious relationship between *PTEN* mutation and survival, but observed a trend for *PTEN* mutation to occur in an older group of patients. In our small series there is no obvious relationship between patient age and the presence of *PTEN* mutation. The *PTEN* gene is now becoming well established as an important tumour suppressor gene in the development of many malignancies. These observations highlight the need for further research to determine its prognostic significance particularly in gliomas.

© Cancer Research Campaign 1999

### ACKNOWLEDGEMENTS

This work was supported by Clatterbridge Cancer Research Trust. We are grateful to the staff of the Histopathology Department and to neurosurgeons Mr T Pigott and Mr P Eldridge from the Neurosurgery Centre, Walton Hospital, Liverpool for help and support during this project.

### REFERENCES

Bigner SH, Mark J, Burger PC, Mahaley MS Jr, Bullard DE, Muhlbaier LH and Bigner DD (1988) Specific chromosomal abnormalities in malignant human gliomas. *Cancer Res* **48**: 405–411

Black PM (1991) Brain tumours (first of two parts). *N Engl J Med* **324**: 1471–1476

Boström J, Cobbers JMJL, Wolter M, Tabatabai G, Weber RG, Lichter P, Collins VP and Reifenberger G (1998) Mutation of the *PTEN* (*MMAC1*) tumor suppressor gene in a subset of glioblastomas but not in meningiomas with loss of chromosome arm 10q. *Cancer Res* **58**: 29–33

Cairns P, Okami K, Halachmi S, Halachmi N, Esteller M, Herman JG, Jen J, Isaacs WB, Bova GS and Sidransky D (1997) Frequent inactivation of *PTEN*/*MMAC1* in primary prostate cancer. *Cancer Res* **57**: 4997–5000

Chiariello E, Roz L, Albarosa R, Magnani I and Finocchiaro G (1998) *PTEN*/*MMAC1* mutations in primary glioblastomas and short-term cultures of malignant gliomas. *Oncogene* **16**: 541–545

Going JJ and Lamb RF (1996) Practical histological microdissection for PCR analysis. *J Pathol* **179**: 121–124

Guldberg P, Thor Straten P, Birck A, Ahrenkiel V, Kirkin AF and Zeuthen J (1997) Disruption of the *MMAC1*/*PTEN* gene by deletion or mutation is a frequent event in malignant melanoma. *Cancer Res* **57**: 3660–3663

Hildebrand J, Dewitte O, Dietrich PY and de Tribolet N (1997) Management of malignant brain tumors. *Eur Neurol* **38**: 238–253

Karlbom AE, James CD, Boethius J, Cavenee WK, Collins VP, Nordenskjold M and Larsson C (1993) Loss of heterozygosity in malignant gliomas involves at least three distinct regions on chromosome 10. *Hum Genet* **92**: 169–174

Kleihues P, Burger PC and Scheithauer B (1993) *Histological Typing of Tumours of the Central Nervous System*. Springer-Verlag: Berlin

Li DM and Sun H (1997) TEP1, encoded by a candidate tumor suppressor locus, is a novel protein tyrosine phosphatase regulated by transforming growth factor β. *Cancer Res* **57**: 2124–2129

Li J, Yen C, Liaw D, Podsypanina K, Bose S, Wang SI, Puc J, Miliaresis C, Rodgers L, McCombie R, Bigner SH, Giovanella BC, Ittmann M, Tycko B, Hibshoosh H, Wigler MH and Parsons R (1997) *PTEN*, a putative protein tyrosine phosphatase gene mutated in human brain, breast and prostate cancer. *Science* **275**: 1943–1947

Liaw D, Marsh DJ, Li J, Dahia PLM, Wang SI, Zheng Z, Bose S, Call KM, Tsou HC, Peacocke M, Eng C and Parsons R (1997) Germline mutations of the *PTEN* gene in Cowden disease, an inherited breast and thyroid cancer syndrome. *Nat Genet* **16**: 64–67

Liu W, James CD, Frederick L, Alderete BE and Jenkins RB (1997) *PTEN*/*MMAC1* mutations and *EGFR* amplification in glioblastomas. *Cancer Res* **57**: 5254–5257

Louis DN (1997) A molecular genetic model of astrocytoma histopathology. *Brain Pathol* **7**: 755–764

Lynch ED, Ostermeyer EA, Lee MK, Arean JF, Ji HL, Dann J, Swisshelm K, Suchard D, MacLeod PM, Kvinnsland S, Tore Gjertsen B, Heimdal K, Lubs H, Møller P and King MC (1997) Inherited mutations in *PTEN* that are associated with breast cancer, Cowden disease and juvenile polyposis. *Am J Hum Genet* **61**: 1254–1260

Marsh DJ, Dahia PLM, Zheng Z, Liaw D, Parsons R, Gorlin RJ and Eng C (1997) Germline mutations in *PTEN* are present in Bannayan–Zonana syndrome. *Nat Genet* **16**: 333–334

Marsh DJ, Coulon V, Lunetta KL, Rocca-Serra P, Dahia PLM, Zheng Z, Liaw D, Caron S, Duboue B, Lin AY, Richardson AL, Bonnetblanc JM, Bressieux JM, Cabarrot-Moreau A, Chompret A, Demange L, Eeles RA, Yahanda AM, Fearon ER, Fricker JP, Gorlin RJ, Hodgson SV, Huson S, Lacombe D, LePrat F, Odent S, Toulouse C, Olopade OI, Sobol H, Tishler S, Woods CG, Robinson BG, Weber HC, Parsons R, Peacocke M, Longy M and Eng C (1998) Mutation spectrum and genotype-phenotype analyses in Cowden disease and Bannayan–Zonana syndrome, two hamartoma syndromes with germline *PTEN* mutation. *Hum Mol Genet* **7**: 507–515

Myers MP and Tonks NK (1997) *PTEN*: sometimes taking it off can be better than putting it on. *Am J Hum Genet* **61**: 1234–1238

Nelen MR, van Staveren WCG, Peeters EAJ, Ben Hassel M, Gorlin RJ, Hamm H, Lindboe CF, Fryns JP, Sijmons RH, Woods DG, Mariman ECM, Padberg GW and Kremer H (1997) Germline mutations in the *PTEN*/*MMAC1* gene in patients with Cowden disease. *Hum Mol Genet* **6**: 1383–1387

Nollau P and Wagener C (1997) Methods for detection of point mutations: performance and quality assessment. *Clin Chem* **43**: 1114–1128

Ohgaki H, Schäuble B, zur Hausen A, von Ammon K and Kleihues P (1995) Genetic alterations associated with the evolution and progression of astrocytic brain tumours. *Virchows Arch* **427**: 113–118

Parsons R (1998) Phosphatases and tumorigenesis. *Curr Opin Oncol* **10**: 88–91

Rasheed BK, McLendon RE, Friedman HS, Friedman AH, Fuchs HE, Bigner DD and Bigner SH (1995) Chromosome 10 deletion mapping in human gliomas: a common deletion region in 10q25. *Oncogene* **10**: 2243–2246

Rasheed BKA, Stenzel TT, McLendon RE, Parsons R, Friedman AH, Friedman HS, Bigner DD and Bigner SH (1997) *PTEN* gene mutations are seen in high-grade but not in low-grade gliomas. *Cancer Res* **57**: 4187–4190

Rhei E, Kang L, Bogomolniy F, Federici MG, Borgen PI and Boyd J (1997) Mutation analysis of the putative tumor suppressor gene *PTEN*/*MMAC1* in primary breast carcinomas. *Cancer Res* **57**: 3657–3659

Risinger JI, Hayes K, Berchuck A and Barrett JC (1997) *PTEN*/*MMAC1* mutations in endometrial cancers. *Cancer Res* **57**: 4736–4738

Sakurada A, Suzuki A, Sato M, Yamakawa H, Orikasa K, Uyeno S, Ono T, Ohuchi N, Fujimura S and Horii A (1997) Infrequent genetic alterations of the *PTEN*/*MMAC1* gene in Japanese patients with primary cancers of the breast, lung, pancreas, kidney and ovary. *Jpn J Cancer Res* **88**: 1025–1028

Sonoda Y, Murakami Y, Tominaga T, Kayama T, Yoshimoto T and Sekiya T (1996) Deletion mapping of chromosome 10 in human glioma. *Jpn J Cancer Res* **87**: 363–367

Steck PA, Ligon AH, Cheong P, Yung WK and Pershouse MA (1995) Two tumor suppressive loci on chromosome 10 involved in human glioblastomas. *Genes Chromosomes Cancer* **12**: 255–261

Steck PA, Pershouse MA, Jasser SA, Yung WKA, Lin H, Ligon AH, Langford LA, Baumgard ML, Hattier T, Davis T, Frye C, Hu R, Swedlund B, Teng DHF and Tavtigian SV (1997) Identification of a candidate tumour suppressor gene, *MMAC1*, at chromosome 10q23.3 that is mutated in multiple advanced cancers. *Nat Genet* **15**: 356–362

Suzuki H, Freije D, Nusskern DR, Okami K, Cairns P, Sidransky D, Isaacs WB and Bova GS (1998) Interfocal heterogeneity of *PTEN*/*MMAC1* gene alterations in multiple metastatic prostate cancer tissues. *Cancer Res* **58**: 204–209

Tashiro H, Blazes MS, Wu R, Cho KR, Bose S, Wang SI, Li J, Parsons R and Ellenson LH (1997) Mutations in *PTEN* are frequent in endometrial carcinoma but rare in other common gynecological malignancies. *Cancer Res* **57**: 3935–3940

Wang SI, Puc J, Li J, Bruce JN, Cairns P, Sidransky and Parsons R (1997) Somatic mutations of PTEN in glioblastoma multiforme. *Cancer Res* **57**: 4183–4186

 *© Cancer Research Campaign 1999*
